# Continuous facial myokymia in multiple sclerosis

**DOI:** 10.1002/ccr3.3135

**Published:** 2020-07-16

**Authors:** Jordan L. Clay, Mauricio F. Villamar

**Affiliations:** ^1^ Department of Neurology University of Virginia Health Systems Charlottesville VA USA; ^2^ Department of Neurology Warren Alpert Medical School of Brown University Providence RI USA; ^3^ Kent Hospital Warwick RI USA

**Keywords:** brainstem, facial myokymia, movement disorders, multiple sclerosis

## Abstract

Facial myokymia is a clinical sign that can occur as a manifestation of demyelinating lesions. As seen in our patient with multiple sclerosis, acute‐onset continuous facial myokymia can be indicative of an active lesion and can have localizing value.

## Graphical abstract

Facial myokymia is a clinical sign that can occur as a manifestation of demyelinating lesions. As seen in our patient with multiple sclerosis, acute‐onset continuous facial myokymia can be indicative of an active lesion and of localizing value.

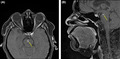

A 54‐year‐old woman with relapsing‐remitting multiple sclerosis (MS) presented with 3 days of continuous facial “quivering” after self‐discontinuing dimethyl fumarate. Examination revealed continuous right perioral myokymia (Video S1). Figure 1 shows her neuroimaging. She received intravenous methylprednisolone. At 1‐month follow‐up, facial myokymia (FM) had resolved and dimethyl fumarate was restarted.

**Figure 1:**
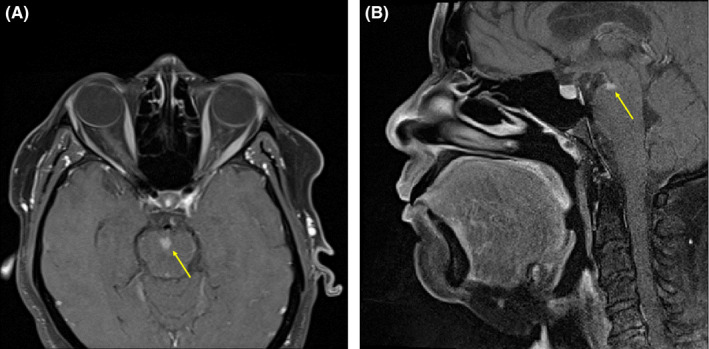
Axial (Panel A) and sagittal (Panel B) postgadolinium T1‐weighted brain MRI revealed a new enhancing lesion in the right superior pons (arrows), ipsilateral to the patient's perioral myokymia

Facial myokymia is an involuntary, abnormal activity consisting of undulating, vermicular movements.^1^, ^2^ FM has been associated with lesions of the postnuclear, postgenu portion of the ipsilateral facial nerve intra‐axially in the dorsolateral pontine tegmentum.^1^ However, many cases show no intra‐axial demyelination of the facial nerve.^2^ In MS patients, FM is usually self‐limited.^2^

## CONFLICT OF INTEREST

None declared.

## AUTHOR CONTRIBUTIONS

JLC: contributed to the case concept and design, acquired the data, interpreted the data, and wrote the manuscript. MFV: contributed to the case concept and design, acquired the data, interpreted the data, and wrote the manuscript.

## Supporting information

Video S1
